# Ankle Sensor-Based Detection of Freezing of Gait in Parkinson’s Disease in Semi-Free Living Environments

**DOI:** 10.3390/s25061895

**Published:** 2025-03-18

**Authors:** Juan Daniel Delgado-Terán, Kjell Hilbrants, Dzeneta Mahmutović, Ana Lígia Silva de Lima, Richard J. A. van Wezel, Tjitske Heida

**Affiliations:** 1 TechMed Centre, Biomedical Signals and Systems Group, Faculty of Electrical Engineering, Mathematics and Computer Science (EEMCS), University of Twente, 7522 NB Enschede, The Netherlands; kjellhilbrants@icloud.com (K.H.); richard.vanwezel@donders.ru.nl (R.J.A.v.W.); t.heida@utwente.nl (T.H.); 2 Department of Biophysics, Donders Institute for Brain, Cognition and Behaviour, Radboud University, 6525 XZ Nijmegen, The Netherlands; dzenetamahmutovic96@outlook.com (D.M.); ana.ligialima@hotmail.com (A.L.S.d.L.); 3 Oneplanet Research Center, Radboud University, 6525 EC Nijmegen, The Netherlands

**Keywords:** deep learning, detection, freezing of gait, Parkinson’s disease, wearable sensors

## Abstract

Freezing of gait (FOG) is a motor symptom experienced by people with Parkinson’s Disease (PD) where they feel like they are glued to the floor. Accurate and continuous detection is needed for effective cueing to prevent or shorten FOG episodes. A convolutional neural network (CNN) was developed to detect FOG episodes in data recorded from an inertial measurement unit (IMU) on a PD patient’s ankle under semi-free living conditions. Data were split into two sets: one with all movements and another with walking and turning activities relevant to FOG detection. The CNN model was evaluated using five-fold cross-validation (5Fold-CV), leave-one-subject-out cross-validation (LOSO-CV), and performance metrics such as accuracy, sensitivity, precision, F1-score, and AUROC; Data from 24 PD participants were collected, excluding three with no FOG episodes. For walking and turning activities, the CNN model achieved AUROC = 0.9596 for 5Fold-CV and AUROC = 0.9275 for LOSO-CV. When all activities were included, AUROC dropped to 0.8888 for 5Fold-CV and 0.9017 for LOSO-CV; the model effectively detected FOG in relevant movement scenarios but struggled with distinguishing FOG from other inactive states like sitting and standing in semi-free-living environments.

## 1. Introduction

Parkinson’s disease (PD) is a progressive neurodegenerative disorder that affects an increasing number of people worldwide [1]. In 2019 alone, approximately 53,000 people were diagnosed with Parkinson’s in the Netherlands [2]. The incidence of PD increases with age, with a significant increase occurring after the age of 65. As life expectancy increases [3], the number of people with PD grows every year and, consequently, the burden of the disease on society.

PD patients experience a wide range of motor symptoms, including balance issues, tremors, bradykinesia, rigidity, and freezing [4]. Freezing of gait (FOG), among all the movement symptoms, is considered one of the most disabling. Often described as “feeling glued to the floor”, FOG occurs in approximately 10% of early-stage PD patients and more than 90% of later-stage patients [5]. FOG often leads to balance impairments and falls [6]. Moreover, FOG is frequently accompanied by non-motor symptoms such as depression, anxiety, and fear of falling (FoF), which are the strongest predictors of low quality of life (QoL), more so than the motor symptoms alone [7,8].

Accurately detecting and tracking FOG episodes can be of valuable help in improving physiotherapy and pharmacotherapy, which are proven methods for reducing FOG episodes [6]. In addition, monitoring FOG features such as frequency, duration, and acuteness together with motor and non-motor symptoms gives physicians more insight into the development and status of the disease in the patient.

Currently, FOG is primarily assessed through subjective patient self-reporting. However, many patients are unable to identify FOG correctly, leading to under-diagnosis. Alternatively, FOG can be objectively diagnosed via a visual gait assessment that experienced clinicians perform during consultations. Yet, the episodic character of FOG makes it difficult for clinicians to assess FOG accurately [9]. Therefore, the accurate and continuous evaluation of FOG in home environments is critical to diagnose and adequately manage FOG. This can be performed using wearable sensors, which enable the assessment of FOG while participants are in FOG-evoking environments and performing daily tasks, such as sitting, walking, lying down, setting up a table, walking outside, or doing chores around the house. These sensors are mostly unobtrusive, portable, and power-efficient, creating the opportunity to measure long-term, enabling the possibility to predict and detect FOG in PD patients throughout the day.

Recent research has shown promising results using wearable sensors to detect early signs of FOG episodes by combining multiple sensors, such as movement, heart rate variability (HRV retrieved from PPG), electroencephalography (EEG), and skin conductivity. Alrichs et al. [10], for example, created an algorithm to detect FOG episodes in patients’ homes using a waist-worn motion capture sensor, detecting the FOG episodes using machine learning (ML) methods such as support vector machine (SVM) on 20 patients. Other methods, such as neural networks (NNs) and deep neuronal networks (DNNs) have also been used in combination with sensors such as EMG and accelerometers, attaining 82.9% sensitivity and 97.3% specificity in detecting FOG across 10 participants [11]. Moreover, ankle sensors have been widely used with other sensors, yet the exclusive use of ankle sensors for FOG detection has been explored only sparingly. Mikos et al. [12] utilized two IMUs placed on both ankles, applying neural networks to data collected during a standardized 7 m timed up and go (TUG) course. Their method achieved a sensitivity of 95.9% and specificity of 93.1% using data from 25 participants. Similarly, Naghavi et al. [13] used accelerometers on both ankles and an Adaptive Synthetic sampling algorithm to analyze a series of daily walking tasks, achieving an accuracy of 97.4% with 18 participants. Naghavi and Wade [14] expanded this approach by incorporating accelerometers and gyroscopes on both ankles in seven participants, employing a transfer learning-based method called the deep gait anomaly detector (DGAD). Tested on walking tasks, this method reported an average sensitivity of 63.0% and a specificity of 98.6%. In contrast, Punin et al. [15] used a single IMU placed on the right ankle, applying a discrete wavelet transform to data collected from highly controlled tasks, including stair climbing and descending, straight walking, and 180° turns. Their method achieved a sensitivity of 86.66% and specificity of 60.61% over seven participants, where only six showed FOG episodes. To date, the study of Punin et al. [15] remains the only one to use data from a single ankle sensor, in this case, in the right ankle for FOG detection, though their tasks were highly structured and controlled.

Recent advances further underscore the potential of wearable sensor systems and machine learning for real-time FOG management. For example, Koltermann et al. [16] introduced GaitGuard—a closed-loop, portable system that achieved a 96.5% true positive rate with an intervention latency of about 378 ms and markedly reduced false positives across 26 participants. Yang et al. [17] compared a two-step Transformer–XGBoost approach and an end-to-end temporal convolutional model for FOG classification in 18 participants, reporting similar performance whether or not additional physiological data were used. Additionally, Slemenšek et al. [18] demonstrated a microcontroller-based system operating at 40 Hz, which reached 95.1% detection accuracy with a 261 ms delay and reduced FOG duration by 45% in nine participants. Together, these studies demonstrate the potential of optimized sensor configurations and deep learning methods for improving FOG detection. However, ensuring they work reliably outside of controlled environments is still a challenge.

Most of these systems struggle to maintain the same performance in home environments, as algorithms created in the laboratory do not address the large variability of human behavior seen in daily life (free-living conditions) and have limited datasets and therefore limited episodes that could most likely impair performance. Furthermore, finding an optimal balance between wearability and performance remains a challenge. O’Day et al. [19] evaluated different IMU configurations for FOG detection and patient preferences. Their study demonstrated the successful adaptation of an intervention for detecting FOG in PD using only a single ankle sensor. The ankle sensor was rated as the most wearable, together with the wrist and lumbar sensors, emphasizing the importance of more portable and simplified systems. However, their detection AUROC was (AUROC = 0.80). This research addresses these gaps by introducing a novel approach that optimizes the accuracy and wearability of FOG detection systems. Specifically, this study aims to develop and test a deep learning (DL) model for detecting FOG episodes in PD patients in a semi-free living environment using a minimal inertial measurement unit (IMU) setup placed at the right ankle, offering a more realistic assessment of FOG detection in naturalistic settings. Compared to other methods used for temporal analysis, such as long short-term memory (LSTM) networks, CNNs can efficiently extract spatiotemporal features while maintaining lower computational complexity, making them more suitable for real-time applications. Unlike LSTMs, which process sequential data step by step and require a memory mechanism, CNNs apply convolutional filters to capture temporal dependencies in parallel, resulting in faster computation and reduced latency [20,21]. Moreover, the real-time detection of freezing episodes during daily life enables the possibility of providing cueing, i.e., preventing or reducing FOG episodes through rhythmic visual, auditory, or somatosensory cues. Cueing is a crucial intervention that helps improve gait recovery and reduces the risk of falls, enhancing patient safety and mobility in daily life [22,23].

## 2. Materials and Methods

### 2.1. Participants

We recruited and measured 24 self-reporting idiopathic PD participants who experience daily episodes of FOG. Inclusion criteria required participants to have a self-reported diagnosis of PD, use levodopa or other PD medication, be 18 years or older, experience FOG episodes daily (as indicated by a response of “Very often, more than once a day” to question 2 of the New Freezing of Gait Questionnaire), have no cognitive or psychiatric impairment as judged by the researcher, and be able to provide informed consent. Exclusion criteria were incapacitating dyskinesias or dystonia, comorbidities causing severe gait impairment (e.g., severe arthrosis or neuropathy), and the usage of advanced therapies such as deep brain stimulation (DBS). The severity and progression of motor and non-motor symptoms were assessed using the Movement Disorder Society’s Unified Parkinson’s Disease Rating Scale section III (MDS-UPDRS III/II) and the Mini-BEST test for balance. Moreover, the participants completed the Montreal Cognitive Assessment, Fall history, and ABC scale questionnaires.

### 2.2. Procedure

Participants were assessed at the eHealth House (eHH) at the TechMed Centre of the University of Twente (Enschede, The Netherlands). The initial part of the measurements was conducted after a withdrawal of levodopa for at least 12 h. By doing so, participants were initially assessed in a clinically defined OFF state, during which FOG occurs more frequently. The activities performed—such as the morning routine, including cleaning, setting out the table for lunch, mopping, making the bed among other daily activities, outdoor walk, and clinical assessments—were repeated in the afternoon after the participant had taken their medication (30 min before lunch), thus entering an ON state. The order of the study procedures performed after medication intake was randomized.

### 2.3. Materials

To address the study’s main endpoint in the semi-free-living condition, data were collected from 17 IMU sensors attached to various body locations, a smartphone application tracking GPS, body acceleration, and angular rate, a smartwatch measuring skin conductance (SC) and heart rate (HR), two additional ankle IMU sensors, insole sensors capturing the center of pressure (COP) and reaction forces, disease severity assessments, and video recordings. However, only the IMU sensor on the right ankle was used for this study. The IMU placed at the right ankle was chosen for its promising performance and patient wearability ratings. Given their proven effectiveness in PD research and similar domains, the model primarily utilizes convolutional neural networks (CNNs) for local pattern recognition tasks [24]. Additionally, O’Day et al. [19] found ankle-mounted sensors to be more comfortable and wearable than waist sensors. Waist sensors, while tracking overall body movement, are prone to displacement and interference from trunk dynamics, making the right ankle sensor a more practical option for long-term monitoring.

Six cameras in several parts of the eHH recorded all measurement sessions, capturing the totality of the participant’s tests and activities. An additional GoPro pointed at the participant’s feet was used to have an extra angle and to record the walk outside, where the other cameras were out of reach. The videos were annotated using the standardized procedure developed by Gilat et al. [25], where the start and end points of an FOG episode were determined based on the definition provided by Nutt et al. [26]. Annotations were performed by two independent trained raters using the open source program ELAN software (The Language Archive, Nijmegen, The Netherlands).

The experiments were run with an Intel^®^ HD Graphics 4600 processor with 3.60 GHz, 28 GB RAM, and 1 GB GPU (Intel Corporation, Santa Clara, California, USA). Preprocessing, building the model, training, and testing were performed in Python 3.12 using TensorFlow 2.16.1 and Keras 3.2.1.

### 2.4. Preprocessing

Initially, all the data were aligned with the video annotations, with the GoPro camera serving as the central reference point. For synchronization, the sensors were tapped five times in front of the camera to be further matched with the signals using ELAN. This procedure was performed at the start of the morning and afternoon sessions. All data were annotated based on the video recordings to differentiate between daily activities such as walking, turning, sitting, standing, laying, and FOG episodes.

Next, the data were processed using a zero-phase third-order Butterworth band-pass filter (0.3–15 Hz) to remove drift and high-frequency noise, while maintaining the frequency ranges relevant to locomotion (0.5–3 Hz) and FOG (3–8 Hz) [18]. The filtered data were then resampled to 60 Hz to standardize sample frequencies across sensors and ensure sufficient resolution for movement analysis [19]. For the CNN model input, data were segmented into 2 s windows.

**Figure 1:**
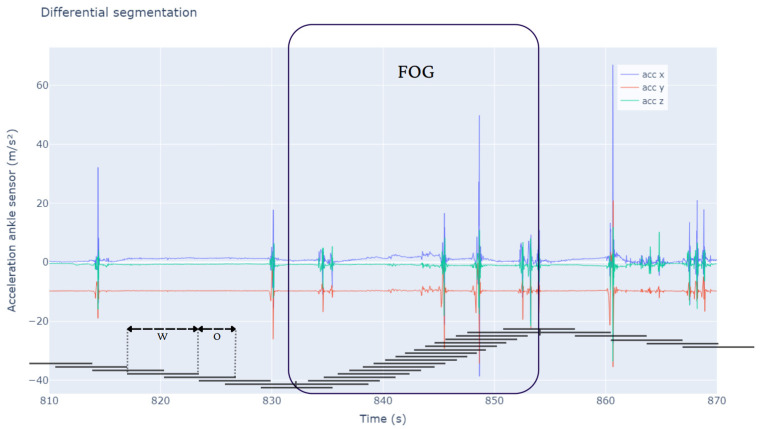
This figure illustrates the differential segmentation approach for balancing FOG and non-FOG data. The large segment labeled ’FOG’ highlights an episodic FOG event, where segmentation intervals overlap by 87.5%. In contrast, adjacent intervals for non-FOG activities overlap by 50%. The x axis shows time, while the y axis indicates the accelerometer of the ankle sensor values across axes. The segmentation method ensures a more balanced data representation despite FOG’s episodic nature using different overlap percentages—87.5% for FOG episodes and 50% for non-FOG activities. W: window length; O: overlap.

Differential segmentation (DS) was employed to address the class imbalance, based on Borzì et al. [27], as illustrated in Figure 1. FOG episodes are rare and brief, needing an 87.5% overlap (0.25 s) to maximize the number of FOG windows and ensure sufficient positive samples for training. In contrast, non-FOG activities, being more frequent and continuous, used a 50% overlap (1 s) to avoid excessive redundancy while maintaining representativeness. Windows were labeled as FOG if 25% of samples (0.5 s) contained FOG, maximizing the number of FOG windows while retaining sufficient training data for the model, and windows with less than 25% were discarded, similarly to the method developed by Klaver et al. [28]. 

The dataset was divided into two datasets, all-activities, which includes all the activities such as walking, turning, standing, sitting, small steps, and transitions, and walking-turning, which isolated walking and turning episodes, which are the activities that show a higher correlation with FOG.

### 2.5. CNN Model Architecture

**Figure 2:**
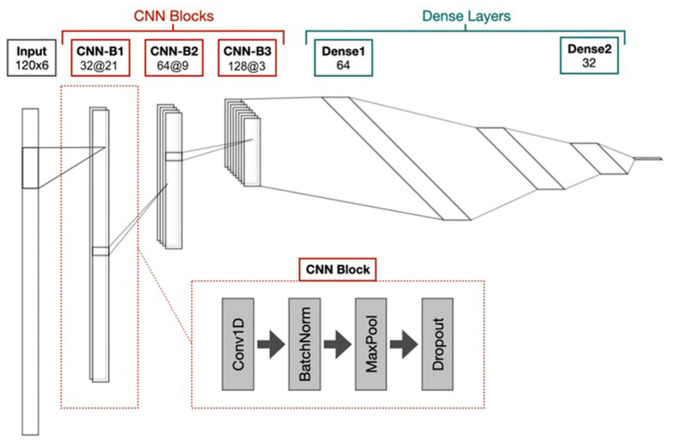
The model processes input data as time series windows, containing 120 samples across 6 channels. The first portion of the model is composed of three consecutive convolutional neural network (CNN) blocks, referred to as CNN-B1, CNN-B2, and CNN-B3. Each CNN block includes a 1D convolutional layer (Conv1D), followed by batch normalization, max pooling, and dropout. The number of filters and kernel size for each block are specified as Filters@Kernel. After the final convolutional block (CNN-B3), the output is flattened and passed into the second half of the model, consisting of three fully connected (dense) layers. The size of these dense layers decreases progressively, starting with 64 units in the first dense layer (Dense1) and reducing to 32 units in the second dense layer (Dense2).

The model architecture is based on established practices in deep learning for time-series data analysis, particularly utilizing CNNs for feature extraction from sensor data [24,29]. The model inputs are pre-processed six-channel IMU data with 2 s windows at 60 Hz. These data include readings from a three-axis accelerometer and a three-axis gyroscope, capturing both acceleration and angular velocity. This configuration enables the model to capture the rich spatiotemporal dynamics of human gait, essential for identifying subtle changes associated with FOG. The model architecture is designed to balance computational efficiency and high performance, consisting of two primary components, CNN, and a multilayer perceptron (MLP) (Figure 2).

Feature extraction (CNN):

Three stacked convolutional blocks (CNN-B1–CNN-B3), each comprising the following:

One-dimensional convolutional layer with ReLU (rectified linear unit) activation: Extracts local temporal features from the input data.

Batch normalization: Stabilizes training and improves convergence by normalizing layer activations.

Max pooling: Reduces dimensionality and extracts the most salient features, increasing robustness to small input variations.

Dropout: Prevents overfitting by randomly deactivating neurons during training, encouraging the network to learn more robust representations.

The kernel size and number of filters in each block are as follows:

CNN-B1: Kernel size = 21, Filters = 32
CNN-B2: Kernel size = 9, Filters = 64
CNN-B3: Kernel size = 3, Filters = 128

This hierarchical structure, with kernels of sizes 21, 9, and 3 across the CNN blocks, allows the model to learn progressively more complex and abstract features, from low-level sensor data to higher-level representations of movement patterns. A kernel size of 21 in the first block covers a wide temporal window, enabling the model to capture broad trends and overall gait cycles. In contrast, a kernel size of 3 in the final block focuses on very short periods, isolating fine-grained, rapid changes in the sensor data that may signal FOG.

Dropout rates also increase with each block (0.5, 0.6, 0.7), further promoting generalization and preventing over-reliance on any single feature. A lower dropout in early layers permits the capture of broad, general patterns, while a higher dropout in deeper layers mitigates over-reliance on specific neurons, thus preventing overfitting. This progressive strategy encourages robust, distributed feature learning across the network.

Classification (MLP):

The flattened output from the final CNN block (CNN-B3) is fed into an MLP. The MLP consists of two fully connected (dense) and regularized layers with decreasing node counts: 64 and 32 with LeakyReLU and ReLU, respectively. This narrowing structure helps condense the extracted features into a lower-dimensional representation suitable for classification. Each dense layer employs a high dropout rate of 0.8 to mitigate overfitting and enhance generalization. The final layer uses a sigmoid activation function to produce a binary output, indicating the presence or absence of FOG within the input window.

### 2.6. Training and Optimization

The model was trained using the Adam optimizer with an initial learning rate of 5e-5 and binary cross-entropy loss. Data shuffling and batching (size 256) were employed before each training epoch to ensure a balanced representation of both classes (FOG and non-FOG) and improve training efficiency.

Epoch lengths were set to 100 for 5-fold cross-validation (5Fold-CV) and 50 for leave-one-subject-out cross-validation (LOSO-CV). In this context, an epoch refers to one complete pass through the entire training dataset during model training. The choice of 100 epochs for the 5Fold-CV was based on empirical observations, where the model required more training iterations to converge due to greater variability in the training data across different folds. In contrast, LOSO-CV was limited to 50 epochs to mitigate overfitting, as training on all subjects except one can lead to faster convergence. We used both 5- 5Fold-CV and LOSO-CV to evaluate model performance comprehensively. 5Fold-CV assesses generalization across different data splits, ensuring robustness across varied samples, while LOSO-CV tests subject-independent generalization by evaluating the performance on unseen individuals. Our primary goal is to assess model effectiveness in detecting FOG while comparing these methods serves as a secondary objective to understand the trade-offs between intra-subject and inter-subject generalization. Stratification was used to have balanced folds. Class weights were implemented to handle the remaining data imbalance. Model development, training, and evaluation were conducted using TensorFlow for Python.

### 2.7. Hyperparameter Optimization

Various hyperparameters, including the number of CNN blocks, kernel sizes, filter counts, dropout rates, and MLP layer sizes, were meticulously fine-tuned and optimized to achieve the best possible balance between accuracy and AUROC (area under the receiver operating characteristic curve) performance.

### 2.8. Performance Metrics

Various performance metrics are employed to assess the performance of predictive models with accuracy, sensitivity, specificity, and the F1-score stands out as the most prevalent.

$$Accuracy=\frac{TP+TN}{TP+TN+FP+FN}$$

$$Sensitivity\;(Recall)=\frac{TP}{TP+FN}$$

$$Specificity=\frac{TN}{TN+FP}$$

$$Precision=\frac{TP}{TP+FP}$$

$$F1-Score=\frac{TP+TP}{TP+TP+FP+FN}$$

$$Weighted\;Precision=\sum_{i}w_{i}\times \frac{TP}{TP+FP}$$

$$Weighted\;F1-Score=\sum_{i}w_{i}\times {F1}_{i}$$

Accuracy quantifies the overall correctness of predictions. Sensitivity, also called true positive (TP) rate, assesses the ability of the model to identify positive instances correctly. Conversely, specificity, also called a true negative (TN) rate, assesses the ability of the model to recognize accurately negative instances. Precision measures the proportion of correctly predicted positive instances out of all predicted positives. It is important in scenarios where false positives (FPs) need to be minimized. F1-score, calculated as the harmonic mean of precision and recall, is particularly advantageous in scenarios characterized by a class imbalance or when minimizing false positives (FP) and false negatives (FN) is imperative, as it offers a comprehensive assessment of a model’s efficacy. Weighted precision and weighted F1-score extend these metrics by computing a weighted average across all classes, where each class’s contribution is proportional to its prevalence in the dataset. Weighted precision ensures that the model’s performance is properly assessed across imbalanced datasets, while the weighted F1-score provides a more balanced evaluation by incorporating both precision and recall in its weighted formulation. Moreover, the AUROC is used to evaluate the model by measuring its ability to discriminate between positive and negative instances across all possible thresholds, ranging from 0 to 1, with a higher value indicating better discrimination ability.

## 3. Results

The study initially included 24 participants with Parkinson’s disease; however, three were excluded due to the absence of FOG episodes. Ultimately, 21 participants remained for analysis. A total of 811 FOG episodes were captured, accounting for 3.61 h of recorded FOG episodes and 67.78 h of activities, including walking, turning, standing, sitting, lying down, and taking small steps (All-Activities). Walking-Turning, as a subset of All-Activities, constituted 14.25 h.

The median age was 74 years and interquartile range (70.0–75.0), indicating an older population typical of this condition (Table 1). Participants had experienced symptoms for of 13 years (8.0–16.0), with a median diagnosis duration of 10.0 years (5.0–13.5). Motor assessments showed variability, with the Hoen and Yahr scores averaging 0.5 (0.0–2.0) and a mode of 2, suggesting a range of disease severity among participants. The median MDS-UPDRS3 scores were 43.0 (25.0–55.0) in the ON state and 51.0 (42.0–63.0) in the OFF state, highlighting the impact of medication on motor function. Balance and gait assessments, as measured by the Mini-BEST test, with median 17.0 (14.0–21.0) in the ON state and 51.0 (42.0–63.0) in the OFF state, underscoring the challenges that participants face even with treatment. The nFOGQ and ABC Scale scores further reflected the participants’ difficulties with mobility and confidence. Cognitive function, assessed by the MOCA, had a median score of 25.0 (23.0–27.0), indicating mild cognitive impairment common in this population. Most participants reported no history of falls, which may reflect the effectiveness of fall prevention strategies or selection bias.

**Figure 3:**
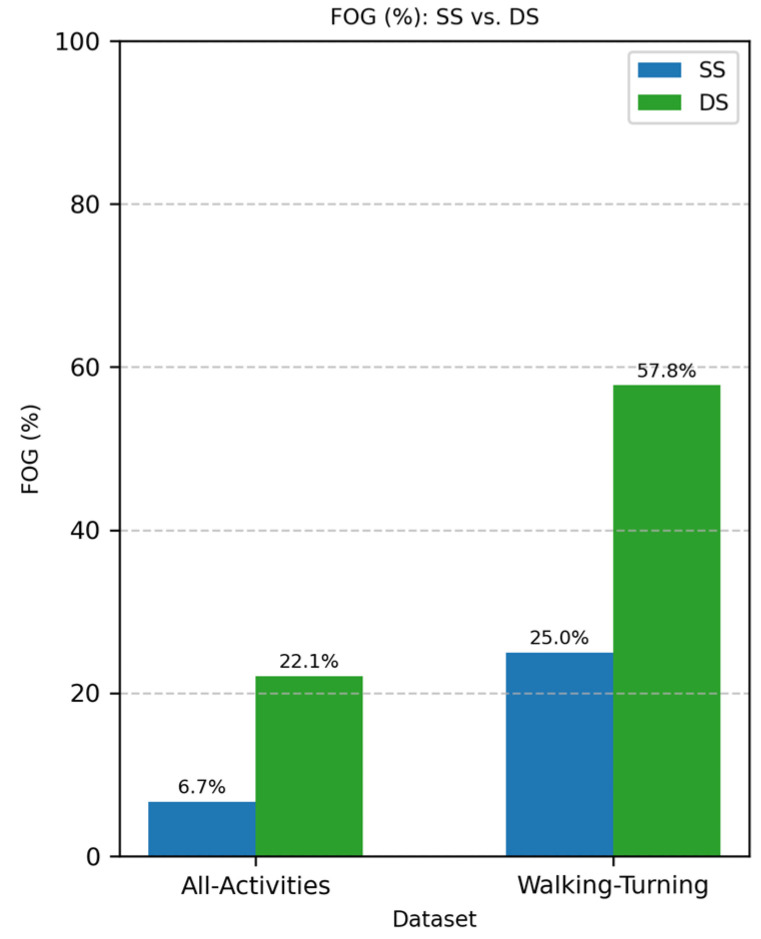
Comparison of FOG proportion with different segmentation methods: standard segmentation (SS, blue) vs. differential segmentation (DS, green). The left side represents All-Activities, while the right shows the Walking-Turning subset. FOG represents freezing of gait episodes, with a higher proportion observed in DS compared to SS for both conditions.

The whole dataset resulted in 3.61 h of FOG episodes, 67.78 h for all the activities (All-Activities), and 14.60 h of walking and turning (Walking-Turning). Differential segmentation improved the dataset balance from 25% of the total FOG windows to 57.8% in the Walking-Turning dataset (Figure 3) from 6.7% to 22.1% in the All-Activities dataset (Figure 3).

### 3.1. Model Performance Metrics

The model has 217,249 trainable parameters ~3.38 M FLOPS and an average inference time per batch of 0.131 s.

Table 2 reports the CNN performance metrics using a 5Fold-CV for the All-Activities and the Walking-Turning datasets, where only walking and turning were used as non-FOG. Table 3 presents the CNN performance subject-wise, using a LOSO-CV and the two datasets.

### 3.2. All-Activities

**Figure 4:**
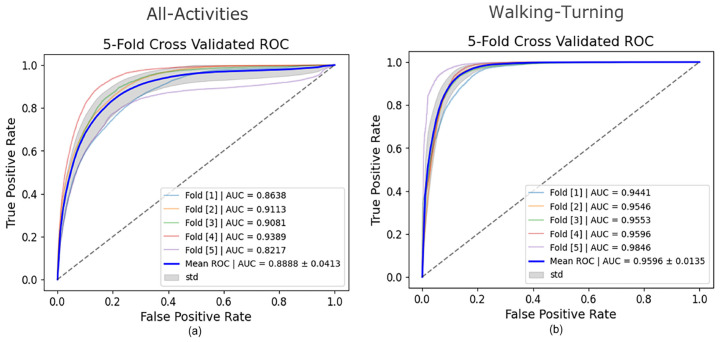
5Fold-CV AUROCs of the All-Activities set vs. FOG (**a**) 5Fold-CV AUROCs of Walking-Turning set vs. FOG (**b**).

**Figure 5:**
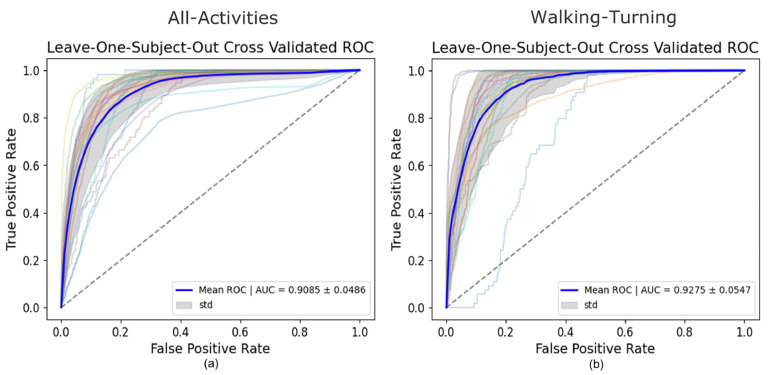
LOSO-CV AUROC of the All-Activities set vs. FOG (**a**) LOSO-CV AUROC of Walking-Turning set vs. FOG (**b**).

**Figure 6:**
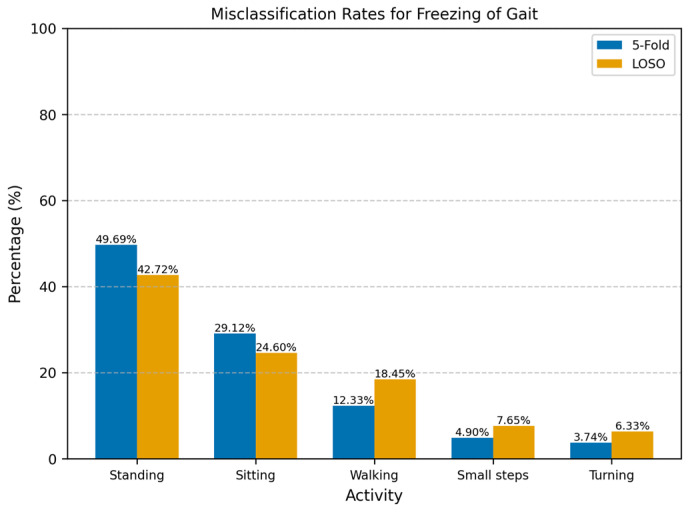
Misclassification rates for freezing of gait across different activities using 5Fold-CV and LOSO-CV. The misclassification rate represents the percentage of incorrectly classified windows for each activity relative to the total number of misclassified windows. Standing was most misclassified as FOG with 49.69%.

For the All-Activities differential segmentation dataset, including augmented data, the model’s performance was also evaluated using 5Fold-CV. The results indicated high overall accuracy and AUROC (Table 2). However, the AUROC values for the individual folds ranged from 0.82 to 0.94 (Figure 4), indicating good overall performance but with more variability and lower performance in all individual folds compared to the Walking-Turning dataset. This suggests that, while the model effectively distinguishes between FOG and non-FOG events across a broader range of activities, its performance can vary depending on the specific subset of activities. When evaluated using LOSO-CV (Table 3), the model maintained a strong AUROC and showed an improved specificity, demonstrating good generalizability; however, the sensitivity was lower than in the 5Fold-CV. Figure 5 compares AUROC values, illustrating the differences between 5Fold-CV and LOSO-CV. Notably, standing had the highest misclassification rate at 49.69% (Figure 6), indicating challenges distinguishing this activity from FOG, which might be due to the static nature of akinetic FOG that could be confused with standing.

### 3.3. Walking-Turning

**Figure 7:**
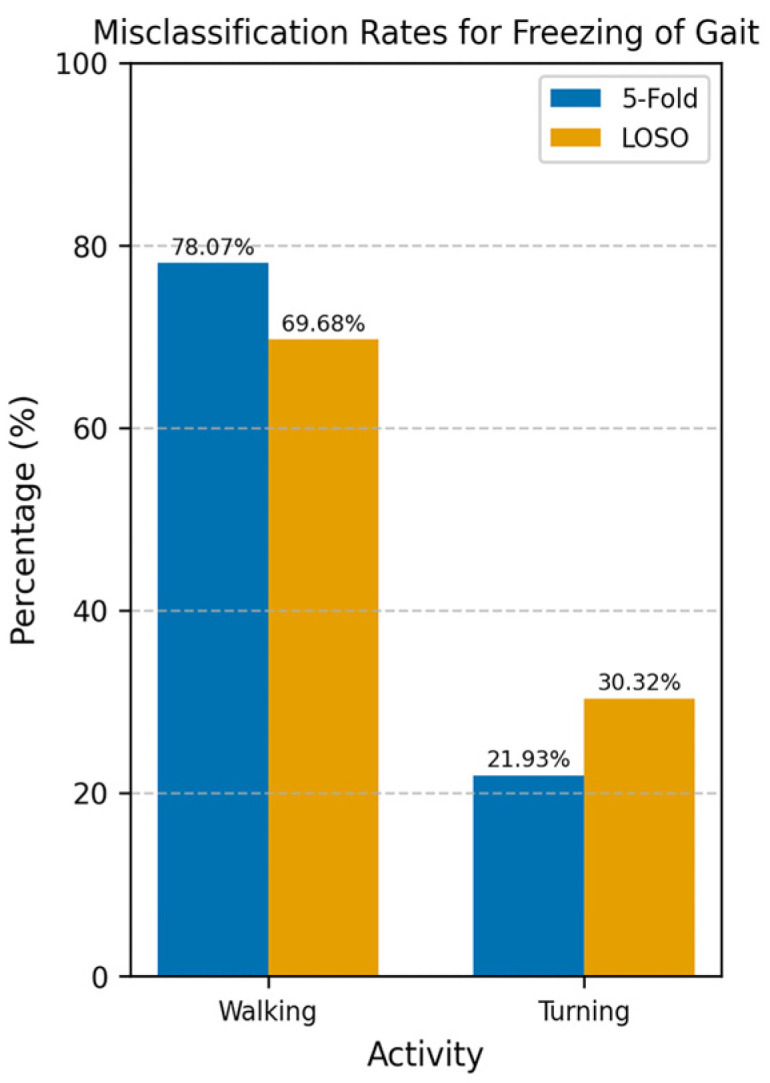
Misclassification rates for freezing of gait across walking and turning using 5Fold-CV and LOSO-CV. The misclassification rate represents the percentage of incorrectly classified windows for each activity relative to the total number of misclassified windows. Walking was most misclassified as FOG with 78%.

The performance of the CNN model on the Walking-Turning differential segmentation dataset was evaluated using a 5Fold-CV approach. The model demonstrated higher performance in this setting than All-Activities, achieving strong performance with increases of 9% in accuracy, 15% sensitivity, and 8% AUROC (Table 2). The model achieved a mean AUROC of 0.96 with a standard deviation of ±0.01 (Figure 4). The AUROC curves for each fold demonstrate high consistency, with AUC values ranging from 0.9441 to 0.9846. This indicates that the model can distinguish between FOG and non-FOG events during walking and turning activities, and its performance is stable across different subsets of data. The number of misclassifications as walking or turning shows walking with the highest number of misclassifications at 78% (Figure 7). Under LOSO-CV (Table 3), the model maintained strong performance, though with slightly lower precision and specificity compared to 5Fold-CV. However, LOSO-CV showed slightly lower precision and specificity compared to 5Fold-CV. Despite this, the model achieved high sensitivity and AUROC, indicating a strong performance in recognizing FOG events across diverse participants.

## 4. Discussion

A CNN-based classification algorithm was developed to detect FOG episodes based on right-ankle IMU measurements in subjects with PD during normal daily activities in a free-living environment. To assess the performance of the model under different conditions, two different datasets were used to train this model separately, one focusing on walking and turning specifically, and another including a wider set of activities, such as walking, turning, standing, sitting, cleaning, taking small steps, and transitions between them. Analyzing both datasets separately gives us insight into how the model works in a controlled and semi-free living environment.

Each dataset was evaluated using 5Fold-CV to assess the models’ overall performance and generalizability. The models achieved strong results, with average AUROCs of 96% for the Walking-Turning dataset and 89% for the All-Activities dataset in 5Fold-CV (Table 2). Furthermore, the Walking-Turning dataset yielded a sensitivity of 96.49% and a specificity of 82.81%, while the All-Activities dataset achieved 81.82% sensitivity and 81.06% specificity. It is important to note that, in clinical settings, the consequences of missing a true FOG episode can be far more severe than the inconvenience of occasional false alarms. Missing a FOG event may lead to falls, injuries, and heightened anxiety among patients. Therefore, even though our model exhibits a slightly lower specificity, the high sensitivity is critical—it ensures that nearly all FOG episodes are detected.

For the scenario where all the activities were included, the differential data segmentation was not enough to reach a balanced training set. However, the test set remained imbalanced since we wanted to assess the model on raw data. The model’s performance was more variable on the All-Activities dataset, with a lower sensitivity (76.42%) and accuracy (83.09%) than on the Walking-Turning dataset. This reduction in sensitivity indicates that the model struggled to detect FOG episodes in a wider set of activities (Figure 6): standing (49.69%) was the activity most misclassified as a FOG episode, and this scenario makes sense since akinetic freezing is very similar to a standing position; the second activity with the most misclassifications was sitting (29.12%), in alignment with the standing position, where there is no apparent movement in the ankle. Despite this, the model achieved a similar specificity to Walking-Turning, suggesting no degradation of the identification of non-FOG activities in a more varied context. Given the imbalanced nature of this dataset, weighted precision and F1-score were particularly relevant, providing a clearer picture of the model’s effectiveness in handling false positives.

The results highlight the model’s strong performance, where activities like walking or turning are mostly performed. However, they also reveal challenges when the model is applied to a broader, more diverse set of activities, one way that could help mitigate these challenges is to use hyperparameter optimization specifically for the All-Activities dataset or the use of transfer learning; however, in our study, we maintained a consistent CNN architecture to enable a direct comparison of performance.

The imbalance in the All-Activities dataset led to a noticeable drop in sensitivity and an overall reduction in performance, as reflected in the weighted F1-Score and Precision.

Focusing on weighted metrics helped us better understand the model’s effectiveness in class imbalances. In this context, the weighted F1-score and precision metrics are essential as they account for the varying significance of each class, offering a clearer picture of the model’s performance across all activity types.

Walking-Turning showed high accuracy (90.90%) (Table 2), overall high performance, and high sensitivity (96.52%), meaning that the model trained with walking and turning events is highly effective in detecting FOG episodes. However, the specificity (83%) was slightly lower than the other metrics, which could lead to greater misclassifications of walking and turning as FOG. The F1-Score was 92.06%, reinforcing the balanced performance of the model to distinguish between FOG episodes and walking and turning, ensuring that a high percentage of FOG episodes are accurately detected. Moreover, a precision of 88.36% indicates that there is still improvement in reducing false positives. An AUROC of 95.96% shows the model’s excellent performance at discriminating between FOG and non-FOG events. Despite lower specificity, this high discrimination between classes reassures the model’s reliability in identifying true FOG episodes across different thresholds.

Table 3 reports the performance metrics for LOSO-CV, showing notably high specificity for All-Activities. However, the model exhibited greater difficulty detecting FOG episodes with unseen participants, likely due to the individual variability of these episodes, leading to a lower sensitivity compared to 5Fold-CV. Despite this, the high specificity reflects the improved recognition of daily activities such as walking, turning, sitting, and standing. Furthermore, the accuracy and AUROC were higher in LOSO-CV, indicating better overall performance. For Walking-Turning, the performance of the LOSO-CV was lower in almost all the metrics compared to 5Fold-CV, which likely derives from the high variability in individual gait and turning patterns. LOSO-CV tests on unseen participants, making it harder for the model to generalize, whereas 5Fold-CV benefits from overlap in participant data, improving performance.

The results from the Walking-Turning dataset are particularly notable compared to previous studies. For instance, Pardoel et al. [30] conducted a comprehensive review of FOG detection and prediction studies, where neural networks reported sensitivity ranges between 72.2% and 99.83% and specificity ranges between 48.4% and 99.96%; these studies normally use several sensors and are based on controlled tasks. In comparison, our sensitivity and specificity results fall within the upper range, demonstrating strong model performance. In addition, Borzì et al. [27] applied a multi-head CNN with a single-waist sensor using data from three different datasets, with a total of 118 PD participants, they reported sensitivity and specificity values of 87.7% and 88.3%, respectively. Our results from the Walking-Turning dataset are higher than these findings in terms of sensitivity (96.49%), although specificity is slightly lower at 83%. Similarly, Camps et al. [31] utilized a single-waist sensor for FOG detection in 21 participants’ homes while performing daily activities, achieving 91.9% sensitivity and 89.5% specificity. While their specificity was higher than ours, our sensitivity outperformed their results, highlighting the robustness of our model for walking and turning tasks.

For ankle sensors, which are relatively few in use, particularly for studies involving a single sensor on the right ankle, Punin et al. [15] remain the only study to employ this setup, reporting a sensitivity of 86.66% and a specificity of 60.61%. This performance was achieved using discrete wavelet transform analysis under controlled conditions, including stair climbing and descending, straight walking, and 180° turns. However, their study assessed only seven participants with one showing no FOG episodes, limiting its generalizability. The proposed model demonstrated superior specificity across all datasets and validation methods, enabling the more efficient detection of daily activities (non-FOG). However, the sensitivity results were mixed. The model performed as expected for the Walking-Turning dataset, exceeding the results reported by Punin et al. [15], whilst performance dropped on the All-Activities dataset, likely due to the high variability in movement patterns and misclassifications during standing and sitting (Figure 6).

These challenges in diverse settings highlight a common issue with single-sensor approaches: differentiating FOG from activities with similar movement. Recent work by Yang et al. [17] has addressed this by integrating additional sensors in 18 participants. In one study, they fused IMU, GSR, and ECG data using both a two-step approach (Transformer-based model plus XGBoost) and an end-to-end temporal convolutional network, achieving F1 scores between 0.728 and 0.771—though the inclusion of physiological data did not significantly improve performance over IMU-only methods (*p* = 0.466–0.887). In another study by Wang et al. [32], combining EEG with accelerometer data slightly improved detection (MCC of 0.211 vs. 0.127–0.139 for single-modal approaches) using data from 15 participants. These findings suggest that, while multimodal systems can show moderate improvements, our single-sensor approach still balances performance and practicality in free-living environments.

Most of the misclassifications on the All-Activities dataset were standing and sitting, making up 78.81% and 67.32% across all the participants for the 5Fold-CV and the LOSO-CV, respectively (Figure 6). These activities involve little to no movement and are often indistinguishable from akinetic freezing episodes. However, the lower rate of misclassification for static activities in the LOSO-CV may be attributed to the unique movement patterns of individual participants during activities such as walking, turning, and taking small steps. To address this challenge, implementing an activity threshold detection system could help filter out periods of inactivity such as lying down, sitting, and standing. Therefore, the model could reduce the number of false positives, since most misclassifications were during periods of inactivity. As implemented before by Borzì et al. [27], the activity threshold method can be implemented using the magnitude of the IMU acceleration, and the activity threshold should be tested using a range of thresholds and assessing its end performance using sensitivity, specificity, and the percentage of discarded windows, while ensuring that valuable information is not removed. Although our primary analysis focused on participants prone to FOG episodes—thereby excluding three participants who did not exhibit any FOG—to mitigate data imbalance, we recognize that including these individuals could add valuable context. Their data may capture alternative walking patterns, potentially enhancing the generalizability of our detection model. However, including such participants introduces significant methodological challenges. Their inclusion would further increase the imbalance in the dataset, which might need more aggressive or diverse data augmentation methods to compensate. Additionally, including participants without FOG would make LOSO-CV impractical, as test sets without FOG events would affect the evaluation of the model’s detection performance.

A different way to address the misclassifications in standing and sitting situations is to add a sensor, such as a psychophysiological sensor including heart rate and skin conductance or insoles, that could measure the participant’s COP, giving more information on the participant’s posture and balance. This may enable us to differentiate between akinetic FOG and standing and sitting. The CNN model addresses the need for solutions in semi-free and free-living environments. Unlike many systems trained and tested under laboratory and clinical conditions, this approach performs well in more variable, real-world scenarios, which are closer to the actual experiences of PD patients.

Furthermore, a minimal sensor system on the ankle maximizes wearability. As O’Day et al. [19] found, a single sensor placed on the ankle showed positive adaptation among participants. This system aligns with the application of cueing devices for real-time detection during real-world scenarios, including a wide range of daily activities. where cues are triggered only when a FOG episode is detected (on-demand cueing). This approach is more effective than continuous cueing, which adds an unnecessary burden and reduces effectiveness for PD patients [33]. Additionally, the computational efficiency of our model—requiring only 217,249 trainable parameters, ~3.38M FLOPS, and an inference time of 0.131 s per batch—ensures that real-time detection can be achieved with minimal processing delay, making it well suited for wearable deployment.

In future applications, the CNN model will be tuned to the data of a single subject, and thus better adjust to individual characteristics, thereby reducing misclassification.

## 5. Conclusions

In conclusion, this study showed the efficacy of the CNN model in detecting Freezing of Gait episodes in PD participants using a single IMU at the ankle in a semi-controlled environment. Unlike prior research relying on multiple sensors or controlled tasks, this study highlights the potential of a simpler, single-sensor setup to detect FOG in diverse movement scenarios, paving the way for practical, real-world applications. These findings highlight the potential of a single-sensor CNN-based approach for FOG detection in semi-controlled and semi-free living environments. Despite the challenges in diverse activity sets, the model’s strong performance in targeted scenarios underscores its potential as a clinical tool or for developing wearable devices for continuous FOG monitoring. Future work should focus on applying an activity threshold to filter out inactive periods, enhancing its applicability in free-living conditions. With further improvements, this approach could be implemented in real-world scenarios to provide timely and accurate detection and enable effective cueing to prevent or mitigate FOG episodes, ultimately enhancing the quality of life for PD patients.

## Figures and Tables

**Table 1:** Summary of demographics, disease history, motor assessments, and cognitive test results for the 21 participants. Continuous variables are presented as the median (interquartile range). Categorical variables are presented as percentages. H&Y: Hoen and Yahr score; MDS-UPDRS III: Unified Parkinson’s Disease Rating Scale, Mini-BEST: Balance Evaluation Systems Test; nFOGQ: new freezing of gait questionnaire, ABC Scale: Activities-specific Balance Confidence Scale; MOCA: Montreal Cognitive Assessment; ON: On medication; OFF: Off medication.

	Variable	
Demographics	Age (years)	74.0 (70.0–75.0)
	Sex (% male)	81
Disease History	Years since symptoms	13.0 (8.0–16.0)
	Years since diagnosis	10.0 (5.0–13.5)
Motor Assessments	H&Y	0.5 (0.0–2.0)
	UPDRS III ON	43.0 (25.0–55.0)
	UPDRS III OFF	51.0 (42.0–63.0)
	Mini-BEST ON	17.0 (14.0–21.0)
	Mini-BEST OFF	25.0 (17.0–27.0)
	nFOGQ	17.0 (13.0–19.0)
	ABC Scale	69.4 (57.5–83.1)
Cognition	MOCA	25.0 (23.0–27.0)

**Table 2:** Performance metrics of the 5Fold-CV using 100 epochs. Values are reported as mean ± standard error.

	All-Activities	Walking-Turning
Accuracy	0.82 ± 0.01	0.91 ± 0.02
Sensitivity	0.81 ± 0.08	0.96 ± 0.02
Specificity	0.82 ± 0.03	0.83 ± 0.03
Precision	0.56 ± 0.08	0.88 ± 0.04
Precision (weighted)	0.86 ± 0.02	0.91 ± 0.02
F1-Score	0.65 ± 0.06	0.92 ± 0.03
F1-Score (weighted)	0.83 ± 0.01	0.91 ± 0.02
AUROC	0.88 ± 0.04	0.96 ± 0.01

**Table 3:** Performance metrics of the leave-one-subject-out CV using 50 epochs. Values are reported as mean ± standard error.

	All-Activities	Walking-Turning
Accuracy	0.87 ± 0.06	0.89 ± 0.07
Sensitivity	0.64 ± 0.23	0.93 ± 0.11
Specificity	0.90 ± 0.05	0.79 ± 0.12
Precision	0.52 ± 0.26	0.70 ± 0.29
Precision (weighted)	0.89 ± 0.07	0.93 ± 0.04
F1-Score	0.55 ± 0.2549	0.77 ± 0.26
F1-Score (weighted)	0.88 ± 0.06	0.90 ± 0.05
AUROC	0.90 ± 0.05	0.93 ± 0.05

## Data Availability

The data will be made available from the authors upon reasonable request.
